# Supplementary material for: Using Behavioural Skills Training with Healthcare Staff to Promote Greater Independence for People Living with Dementia: A Randomised Single-Case Experimental Design
Source: Behav Sci (Basel). 2025 Jun 26;15(7):870. doi: 10.3390/bs15070870 (PMC12292436; doi:10.3390/bs15070870)
Supplement: Supplementary file 1 [file behavsci-15-00870-s001.zip › behavsci-3549266-supplementary.pdf]

Table S1: Within and between condition visual analysis data for Activity of Daily Living (ADL)1: assistance to stand.

Within Condition Analysis						
**ADL1**	P3<br>BL	P3<br>INT	P1<br>BL	P1<br>INT	P2<br>BL	P2<br>INT
Mean	51.67	82.77	36.8	80	40.71	77
Median	55	85	35	75	45	75
Range	45-55	65-100	34-45	65-100	35-45	65-85
% data Level Stability Envelope	3:3<br>100%	6:9<br>66.6%	4:5<br>80%	3:7<br>42.8%	7:7<br>100%	5:5<br>100%
Relative Level Change	45→ 55<br>+10	75→92.5<br>+17.5	34.5→ 40<br>+5.5	75→85<br>+10	45→45<br>0	70→85<br>+10
Absolute Level Change	+10<br>Improving	+35<br>Improving	+11<br>Improving	+25<br>Improving	-10<br>Deteriorating	+20<br>Improving
% data Trend Stability Envelope	3:3<br>100%	7:9<br>77%	5:5<br>100%	7:7<br>100%	7:7<br>100%	5:5<br>100%
Split Middle method Trend Direction	Acceler	Acceler	Acceler	Acceler	Level	Acceler
Stable/ Variable	Stable	Variable	Stable	Stable	Stable	Stable

Between Condition Analysis			
**ADL1**	P3<br>BL-INT	P1<br>BL-INT	P2<br>BL-INT
Trend Direction	Accelerating to Accelerating	Accelerating to Accelerating	Level to Accelerating
Deteriorating/Improving	Improving to Improving	Improving to Improving	Deteriorating to Improving
Stable or Variable	Stable to Variable	Stable to Stable	Stable to Stable
Relative Level Change	54→74<br>+20	39.5→74<br>+34.5	44→70<br>+26
Absolute Level Change	+10	+30	+30
Median Level Change	+30	+40	+30
Mean Level Change	+31.13	+43.2	+36.29
PND	100%	100%	100%
POD	0	0	0

Table S2: Within and between condition visual analysis data for Activity of Daily Living (ADL)2: assistance with drinking.

Within Condition Analysis						
**ADL2**	P1<br>BL	P1<br>INT	P3<br>BL	P3<br>INT	P2<br>BL	P2<br>INT
Mean	73.33	92.22	36	82.86	60	90
Median	70	90	40	80	60	100
Range	70-80	90-100	30-40	70-100	50-70	70-100
% Level<br>Stability Envelope	3:3<br>100%	8:9<br>88.89%	5:5<br>100%	6:7<br>85.71%	7:7<br>100%	3:5<br>60%
Relative Level Change	70→70<br>0	90→100<br>+10	35→35<br>0	70→90<br>+20	60→50<br>-10	75→100<br>+25
Absolute Level Change	0<br>Zero	+30<br>Improving	0<br>Zero	+20<br>Improving	-10<br>Deteriorating	+30<br>Improving
% data Trend<br>Stability Envelope	3:3<br>100%	9:9<br>100%	5:5<br>100%	7:7<br>100%	7:7<br>100%	5:5<br>100%
Split Middle-Method<br>Trend Direction	Level	Acceler	Level	Acceler	Decelerating	Aceler
Stable/<br>Variable	Stable	Stable	Stable	Stable	Stable	Stable

Between Condition Analysis			
**ADL2**	P1<br>BL-INT	P3<br>BL-INT	P2<br>BL-INT
Trend<br>Direction	Level to<br>Accelerating	Level to<br>Accelerating	Decelerating to<br>Accelerating
Deterioratin<br>g/Improving	Zero to Improving	Zero to Improving	Deteriorating to<br>Improving
Stable or<br>Variable	Stable to Stable	Stable to Stable	Stable to Stable
Relative<br>Level<br>Change	70→90<br>+20	35→70<br>+35	50→75<br>+25
Absolute<br>Level<br>Change	0	+40	+20
Median<br>Level<br>Change	+20	+40	+40
Mean Level<br>Change	+18.89	+46.86	+30
PND	88.9%	100%	80%
POD	11.1%	0	20%

P1=participant 1; P2=participant 2; P3=participant 3; BL=baseline; INT=intervention; Acceler=accelerating; PND=percentage of non-overlapping data; POD=percentage of overlapping data.

Table S3: Within and between condition visual analysis data for Activity of Daily Living (ADL)3: assistance to brush teeth.

Within Condition Analysis						
**ADL3**	P3<br>BL	P3<br>INT	P2<br>BL	P2<br>INT	P1<br>BL	P1<br>INT
Mean	52	93.5	53	97.8	77	100
Median	56	100	53	100	78	100
Range	44-56	78-100	50-56	89-100	72-78	100-100
% data Level Stability Envelope	3:3<br>100%	4:6<br>66.66%	4:4<br>100%	5:5<br>100%	6:6<br>100%	3:3<br>100%
Relative Level Change	44→56<br>+12	83→100<br>+17	53→53<br>0	94.5→100<br>+5.5	78→78<br>0	100→100<br>0
Absolute Level Change	+12<br>Improving	+22<br>Improving	+6<br>Improving	+11<br>Improving	0<br>None	0<br>None
% data Trend Stability Envelope	3:3<br>100%	5:6<br>83%	4:4<br>100%	5:5<br>100%	6:6<br>100%	3:3<br>100%
Trend Direction	Acceler	Acceler	Level	Acceler	Level	Level
Stable/Variable	Stable	Stable	Stable	Stable	Stable	Stable

Between Condition Analysis			
**ADL3**	P3<br>BL-INT	P2<br>BL-INT	P1<br>BL-INT
Trend Direction	Accelerating to Accelerating	Level to Accelerating	Level to Level
Deteriorating/Improving	Improving to Improving	Improving to Improving	None to None
Stable or Variable	Stable to Stable	Stable to Stable	Stable to Stable
Relative Level Change	56→83<br>+27	53→94.5<br>+41.5	78→100<br>+22
Absolute Level Change	+22	+33	+22
Median Level Change	+44	+47	+22
Mean Level Change	+41.5	+44.8	+23
PND	100%	100%	100%
POD	0%	0%	0%

P1=participant 1; P2=participant 2; P3=participant 3; BL=baseline; INT=intervention; Acceler=accelerating; PND=percentage of non-overlapping data; POD=percentage of overlapping data.
